# Some novel concepts of intuitionistic fuzzy directed graphs with application in selecting a suitable place for opening restaurant

**DOI:** 10.1016/j.heliyon.2024.e33950

**Published:** 2024-07-05

**Authors:** Waheed Ahmad Khan, Khadija Ali, Amna Fida, Muhammad Asif, Hai Van Pham, Quoc Hung Nguyen, Thanh Trung Le, Le Phuc Thinh Tran

**Affiliations:** a Division of Science and Technology, Department of Mathematics, University of Education Lahore, Attock Campus, Attock 43600, Pakistan; b Division of Science and Technology, Department of Computer Science, University of Education Lahore, Attock Campus, Attock 43600, Pakistan; c School of Information and Communication Technology, Hanoi University of Science and Technology, Hanoi, Viet Nam; d School of Business Information Technology, University of Economics, Ho Chi Minh City, Viet Nam

**Keywords:** Intuitionistic fuzzy directed graphs, Effective arcs, Domination in intuitionistic fuzzy directed graphs, Minimal dominating sets, Domination number

## Abstract

In this manuscript, we first initiate several types of effective arcs of intuitionistic fuzzy directed graphs, followed by discussions on different types of dominations in intuitionistic fuzzy directed graphs and their application in decision-making. The notion of dominations in fuzzy graphs, fuzzy directed graphs, intuitionistic fuzzy graphs and picture fuzzy graphs have been extensively discussed in the literature. Thus, the work presented in our study is two-fold: on one side, it extends the notion of domination in fuzzy directed graphs, while on the other side, it fills the gap existing in the literature. Initially, we introduce several types of effective arcs like semi-*λ* effective arc, semi-*ν* effective arcs in intuitionistic fuzzy directed graphs. Subsequently, we initiate the notions of dominations and domination numbers for several types of intuitionistic fuzzy directed graphs based on these effective arcs. We present numerous significant characterizations of dominations in intuitionistic fuzzy directed graphs by utilizing minimal dominating sets. Furthermore, we investigate the minimal dominating sets and domination numbers of intuitionistic fuzzy-dipaths and dicycles, and provide various fascinating results. Finally, by utilizing the concepts presented in this study, we provide the algorithm for the solution of the problem related to decision-making regarding the opening of restaurants in various areas of the different cities.

## 1 Introduction

The notion of fuzzy sets (FSs) was firstly initiated by Zadeh [1] in 1965. FSs found more flexible compared to the classical sets. Due to its nature, numerous applications of FSs have been explored in various fields such as computer science, management sciences, artificial intelligence, decision-making etc. It has been observed that fuzzy logic manipulates complex types of information more efficiently than the classical logic. As the FSs are flexible, many extensions of FSs have been initiated. In this regard, first generalization of FSs termed IVFSs (depicted in Table 1) was explored by Zadeh [2]. In IVFSs, the membership degree was considered as a subinterval of [0,1], rather than a particular number from [0,1], as in case of FSs. However, the idea of non-membership value was not addressed in either FSs or IVFSs. Atanassov [3] added the concept of non-membership value and coined the new term “intuitionistic fuzzy sets (IFs)”. In FSs, the membership degree of an element was considered within the interval [0,1], with the nonmembership degree was defined as one minus the degree of membership. In contrast, in IFSs both degrees were regarded in different ways having the restriction that sum of these degrees should not exceed 1.

**Table 1:** List of Abbreviations.

*Names*	*Abbreviations*
Fuzzy sets	FSs
Membership degree	MD
Non membership degree	NMD
Interval valued fuzzy sets	IVFSs
Intuitionistic fuzzy sets	IFs
Fuzzy graphs	FGs
Intuitionistic fuzzy graphs	IFGs
Picture fuzzy graphs	PFGs
Fuzzy directed graphs	FDGs
Intuitionistic fuzzy directed graph	IFDGs
Dominating sets	DSs
Minimal independent sets	MISs
Minimal dominating sets	MDSs
Domination numbers	DNs
Effective neighborhood	ENbhd
Closed neighborhood	CNbhd

From past few decades, graph theory become a useful tool to model the real-life problems related to many fields of sciences. Different types of graphs and the concepts related to them have been applied to various research areas such as organizing and structuring of different patterns, networking, decision-making etc. Alternatively, to address real-life issues with uncertainties, the fuzzy logic developed into more useful and significant approach. Recently, different kind of issues with uncertainties have been modeled through fuzzy graphs (FGs). The idea of fuzzy graphs was initiated by Rosenfeld [4]. FGs are proven more effective and flexible as compared to the classical graphs. Many applications of FGs in different fields of sciences have been investigated because of its flexible nature. Bhattacharya [5] introduced several new terms in the theory of FGs. In [6], many advanced operations were introduced. Complement of FGs was initiated in [7]. The concepts of average connectivity in FGs were discussed by Poulik et al. [8]. Many researchers have introduced various concepts of classical graphs in the field of FGs. Overall, FGs played a key role in numerous fields such as social sciences, modeling road networks etc. Ubaid ur Rehman [9] introduced some aggregation operators under complex fuzzy environment and applied towards multi-attribute decision-making. Jana et al. [10] initiated the notion of dombi operators under pythagorean fuzzy information and provided applications in multi-attribute decision making. Mahapatra et al. [11] initiated the applications of edge coloring of FGs. The concepts of FDGs (see Table 1) was presented by the Mordeson and Nair in [12]. FDGs were further investigated in [13]. The notion of bipolar-FDGs with application in decision-making was explored in [14]. The generalized form of FGs called IFGs(see Table 1) was presented in [15]. Similarly, the concepts of complex IFGs with application in networking was presented in [16]. Rashmanlou et al. [17] introduced the notion of IFGs with categorical properties. The notion of domination in IFGs by strong arcs and effective arcs was discussed by [18]. Akram et al. [19] initiated the concepts of competition graphs under complex intuitionistic fuzzy environment. Numerous new concepts related to IFGs like IF-hypergraphs, strong-IFGs, IF-cycles and IF-tree were initiated in [20], [21], [22]. Ali et al. [23] introduced some complex intuitionistic fuzzy power interaction aggregation operators and applied in decision-making techniques. Similarly, the concepts of intuitionistic fuzzy directed graph (IFDGs) with application in decision support system was initiated in [24]. An interval-valued IFDG matrix approach in analyzing roadblocks was investigated in [25]. Nithyanandham et al. [26] introduced energy based bipolar IFDG and provided its applications in decision-making.

The term domination has a significant importance in a classical graphs. Many researchers introduced various forms of dominations in graphs like double Roman domination [27], paired domination [28], broadcast domination [29], triple Roman domination [30], outer-convex domination [31] etc. Subsequently, many terms related to dominations of classical graphs have been shifted to FGs. In this context, by utilizing effective arcs the term domination in FGs was studied by Somasundaram et al. [32]. Shanmugam et al. [33] initiated the concept of bridge domination in FGs. The concepts of covering and paired domination in IFGs was initiated by Sahoo et al. [34]. Domination in FDGs was discussed in [35]. Recently, in [36] the terms broadcasts and dominating broadcasts in FGs were introduced, with applications in transportation model. Parvathi et al. [37] initiated the concepts of dominations in IFGs. In the theory of IFGs, the term double domination was introduced by Nagoorgani et al. [38]. The idea of double domination and regular domination in intuitionistic fuzzy hypergraph was introduced by Sri et al. [39]. The term inverse domination in IFGs was discussed by Stephan et al. [40]. The concept of total perfect and total efficient domination in intuitionistic fuzzy graphs were discussed by Hameed et al. [41]. The term domination in intuitionistic fuzzy incidence graph was introduced by Sadati et al. [42]. The concept of strong pair domination number in intuitionistic fuzzy influence graphs with application for the selection of hospital having the optimal medical facilities was initiated by Rehman et al. [43]. Recently, we (the authors with Guan, Shafi and A. Khan) initiated the notion of domination in IFDGs which is based on strong arcs [44].

In this study, we provide several new concepts of dominations in IFDGs by utilizing the concepts of different types of effective arcs. We provide a direct extension of domination in DFGs on one side and fill the existing gap in the literature on the other. We begin our study by introducing the concepts of various types of effective arcs including semi-*λ* effective arc, semi-*ν* effective arcs etc in IFDGs. Then, we initiate the concepts of domination and domination number of several types of IFDGs based on these effective arcs. Sequentially, we initiate the notion of dominating set (DS), MIS (see Table 1) and MDS. By utilizing minimal dominating sets, we discuss numerous significant characterizations related to domination in IFDG. Furthermore, we investigate the minimal dominating sets (MDSs) and domination numbers (DN) of IF-dipaths and dicycles, yielding various fascinating results. Finally, by using the concepts of domination in IFDGs, we provide the solution to the problem of opening restaurants in several areas of the city.

**Motivations and Novelty.**

In IFDGs, the degree of membership and non-membership of the directed edges are considered that is why it has an extensive domain in comparison with the FDGs. Furthermore, the terms domination in FGs [32], IFGs [37] and PFGs [45] have been established which motivated us to fill the gap in the literature by introducing the concepts of dominations in IFDGs and presents its application towards decision. The summary of the novelty of our work is given below.

1. Firstly, we introduced various types of useful effective arcs like semi-*λ* effective arc, semi-*ν* effective arcs etc in IFDGs. We characterize these terms by providing suitable examples.
2. We investigate the notions of dominating set (SCDS), minimal dominating set (MDS) and domination number of some IFDGs.
3. We introduce the concepts of IF-dipath and IF-dicycle in IFDG and also discuss their properties related to dominations.
4. Lastly, we provide its application towards decision-making.

This manuscript has six sections. In section 2, we provide few necessary terminologies regarding FSs, FGs and their extensions. In section 3, we initiate several types of effective arcs of IFDGs and discuss dominations in IFDGs based on these terms. In section 4, we give the solution of the problem in decision-making which ensures the effectiveness of domination in IFDGs. Section 5 includes the comparative study. Finally, section 6 comprises of concluding remarks and future perspectives of our work.

## 2 Preliminaries

Definition 2.1 [1] A pair (λ,Z) is known as fuzzy set, where *Z* is a nonempty set and λ:Z⟶[0,1] represents the membership function.

Definition 2.2 [46] We can describe an intuitionistic fuzzy set (IFS) *S* on set *Z* as follows.

$$S=\{(s,\lambda_{S}(s),\nu_{S}(s):s\in Z)\}$$

where $\lambda_{S}(s)\in [0,1]$ is the membership degree of s∈S, $\nu_{S}(s)\in [0,1]$ represents the non-membership degree of s∈S satisfying $0\leq \lambda_{S}(s)+\nu_{S}(s)\leq 1$, for all s∈Z.

Definition 2.3 [4] A pair $\overset{˘}{G}=(X,Y)$ is a fuzzy graph (FG), where $X=\{\psi_{X}\}$ and $Y=\{\psi_{Y}\}$ with $\psi_{X}:U\to [0,1]$ and $\psi_{Y}:U\times U\to [0,1]$, with $\psi_{Y}(w,x)\leq \psi_{X}(w)\wedge \psi_{X}(x)$.

Definition 2.4 [15] A pair $G^{⁎}=(S,T)$, where $S=\{\lambda_{S},\nu_{S}\}$, $T=\{\lambda_{T},\nu_{T}\}$, is an IFG on *U*, where

(*i*) $\lambda_{S}:U\to [0,1]$, and $\nu_{S}:U\to [0,1]$ are the degree of membership and non-membership of all element w∈U, respectively, such that $0\leq \lambda_{S}(w)+\nu_{S}(w)\leq 1$, for all w∈U, and

(ii)$\lambda_{T}:W\subseteq U\times U\to [0,1]$ and $\nu_{T}:W\subseteq U\times U\to [0,1]$ satisfying $\lambda_{T}(w,x)\leq min\{\lambda_{S}(w),\lambda_{S}(x)\}$ and $\nu_{T}(w,x)\geq max\{\nu_{S}(w),\nu_{S}(x)\}$ with $0\leq \lambda_{T}(w,x)+\nu_{T}(w,x)\leq 1$, for all (w,x)∈U×U. Here, *S* is the intuitionistic fuzzy node set, and *T* is the intuitionistic fuzzy arc set of $G^{⁎}$.

Definition 2.5 [32] Let $\overset{˘}{G}=(X,Y)$, where $X=\{\psi_{X}\}$ and $Y=\{\psi_{Y}\}$ is a FG of a crisp graph *G*. For w,x∈X, we say *w* dominates *x* in $\overset{˘}{G}$, if $\psi_{(}wx)=\psi_{X}(w)\wedge \psi_{X}(x)$.

Definition 2.6 [32] A subset $X_{1}$ of *X* is the DS in $\overset{˘}{G}$, if for each $w\in X_{1}$ there is $x\in U-X_{1}$ with *w* dominates *x*. A DS $X_{1}$ of a FG $\overset{˘}{G}$ is a MDS, if $X_{1}$ doesn't contain any proper DS of $\overset{˘}{G}$. The DN(from Table 1) of $\overset{˘}{G}$ is the minimum (fuzzy) cardinality of a DS in $\overset{˘}{G}$.

Definition 2.7 [35] Let ${\overset{ˆ}{G}}_{D}$ = (U,B) be a simple directed graph, where *U* is a nonempty finite set and B={(w,x):w,x∈U,w≠x}. A pair ${\overset{˜}{G}}_{D}$ = $\left(\psi_{X},\psi_{Y}\right)$ is fuzzy digraph (FDG), where $\psi_{X}:U⟶[0,1]$ and $\psi_{Y}:B⟶[0,1]$ with $\psi_{Y}((w,x))\leq \psi_{X}(w)\wedge \psi_{X}(x)$, for all w,x∈U.

Definition 2.8 [35] Let ${\overset{ˆ}{G}}_{D}=(U,B)$ be an hidden digraph, where *U* is the set of vertices and *B* represents the set of directed edges. Then ${\overset{˜}{G}}_{D}=(\psi_{U},\psi_{B})$ is the fuzzy directed graph of ${\overset{ˆ}{G}}_{D}$, where $\psi_{U}=\{\psi_{U}(w):w\in U\}$ is the set of vertices (nodes), and $\psi_{B}=\{\psi_{B}((w,x)):w,x\in U\}$ represents the fuzzy directed edges from $\psi_{U}(w)$ to $\psi_{U}(x)$ in a fuzzy digraph ${\overset{˜}{G}}_{D}$.

Definition 2.9 [35] Let ${\overset{ˆ}{G}}_{D}=(U,B)$ be an hidden digraph of a FDG ${\overset{˜}{G}}_{D}=(\psi_{U},\psi_{B})$. Then the arc (w,x)∈B is known as effective arc of ${\overset{˜}{G}}_{D}$, if$\psi_{B}(w,x)\leq \psi_{U}(w)\wedge \psi_{U}(x)$, for all w,x∈U.

Definition 2.10 [24] A pair ${\overset{˙}{G}}_{D}$ = (A,B) is an intuitionistic fuzzy digraph (IFDG) of a digraph ${\overset{ˆ}{G}}_{D}$ = (V,E), where *A* = $\left(\lambda_{A},\nu_{A}\right)$ is an IFS on *V* and $B=(\lambda_{B},\nu_{B})$ represents an intuitionistic fuzzy relation on *V* such that $\lambda_{B}(yz)\leq min\{\lambda_{A}(y),\lambda_{A}(z)\}$; $\nu_{B}(yz)\geq max\{\nu_{A}(y),\nu_{A}(z)\}$, and $0\leq \lambda_{B}(yz)+\nu_{B}(yz)\leq 1$, for all y,z∈V.

## 3 Domination in IFDGs through effective arcs

Firstly, we introduce some types of effective edges in IFDG such as semi-*λ* effective arcs, semi-*ν* effective arcs etc. Afterwards, we initiate the notion of domination in IFDG by utilizing these arcs. In this context, we discuss several useful characteristics of IFDG related to dominations. We also provide some fascinating results related to DS and MDS of IFDGs. Lastly, we present the terms IF-dipath and IF-dicycle of IFDG and explore their MDSs, DNs etc. We also present some interesting results related to IF-dipath and IF-dicycle of IFDG.

Throughout our discussions, ${\overset{˙}{G}}_{D}=(A,B)$ will represent an IFDG of an hidden diagraph ${\overset{ˆ}{G}}_{D}$ = (V,E), where *A* = $\left(\lambda_{A},\nu_{A}\right)$ and $B=(\lambda_{B},\nu_{B})$. We begin our discussion with the definition of an effective arc in an IFDG as follows.

Definition 3.1 An arc (y,z) in an IFDG ${\overset{˙}{G}}_{D}$ is an effective arc, if $\lambda_{B}(y,z)=min\{\lambda_{A}(y)$,

$\lambda_{A}(z)\}$ and $\nu_{B}(y,z)=max\{\nu_{A}(y),\nu_{A}(z)\}$ such that $0\leq \lambda_{B}(y,z)+\nu_{B}(y,z)\leq 1$, for all (y,z)∈B.

Now we present the definitions of the corresponding effective arcs that are semi *λ* -effective arc and semi *ν* -effective arc in an IFDG.

Definition 3.2 An arc (y,z) is known as semi *λ* -effective arc of the IFDG ${\overset{˙}{G}}_{D}$, if $\lambda_{B}(y,z)=min\{\lambda_{A}(y),\lambda_{A}(z)\}$ and $\nu_{B}(y,z)\neq max\{\nu_{A}(y),\nu_{A}(z)\}$ with $0\leq \lambda_{B}(y,z)+\nu_{B}(y,z)\leq 1$, for all (y,z)∈B.

Definition 3.3 An arc (y,z) is known as semi *ν* -effective arc of IFDG ${\overset{˙}{G}}_{D}$, if $\lambda_{B}(y,z)\neq min\{\lambda_{A}(y)$,

$\lambda_{A}(z)\}$ and $\nu_{B}(y,z)=max\{\nu_{A}(y),\nu_{A}(z)\}$ such that $0\leq \lambda_{B}(y,z)+\nu_{B}(y,z)\leq 1$ for all (y,z)∈B.

**Figure 1:**
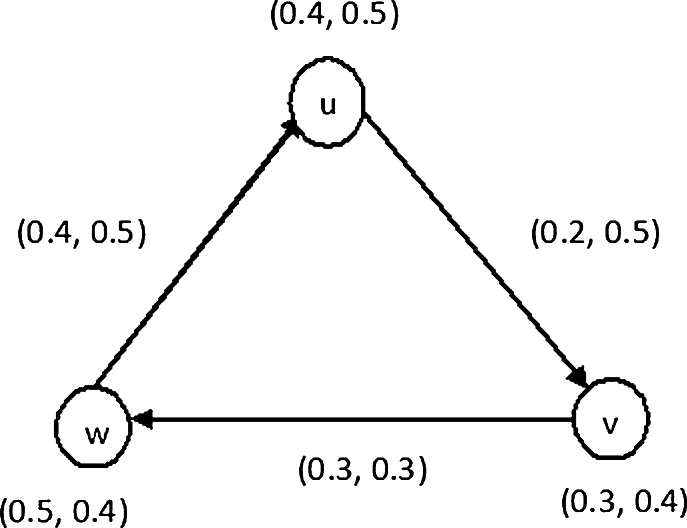
IFDG.

In Example 3.4, we identify the effective arcs in an IFDG given in Fig. 1.

Example 3.4 Refer to IFDG shown in Fig. 1, we have the followings.

(*i*) Consider the arc (u,v), here $\lambda_{B}(u,v)=min\{\lambda_{A}(u),\lambda_{A}(v)\}=min\{0.4,0.3\}=0.3\neq 0.2$ and $\nu_{B}(u,v)=max\{\nu_{A}(u),\nu_{A}(v)\}=max\{0.5,0.4\}=0.5$. Therefore an arc (u,v) is non-effective arc but arc (u,v) is semi *ν* -effective arc.

(ii) Let the arc (v,w), here $\lambda_{B}(v,w)=min\{\lambda_{A}(v),\lambda_{A}(w)\}=min\{0.3,0.5\}=0.3$ and $\nu_{B}(v,w)=max\{\nu_{A}(v),\nu_{A}(w)\}=max\{0.4,0.4\}=0.4\neq 0.3$. Thus the arc (v,w) is non-effective arc but arc (v,w) is semi *λ* -effective arc.

(iii) Let the arc (w,u), here $\lambda_{B}(w,u)=min\{\lambda_{A}(w),\lambda_{A}(u)\}=min\{0.5,0.4\}=0.4$ and $\nu_{B}(w,u)=max\{\nu_{A}(w),\nu_{A}(u)\}=max\{0.4,0.5\}=0.5$. Hence arc (w,u) is an effective arc.

In Definition 3.5, we present the notions of effective neighborhood (ENbhd) and closed neighborhood (CNbhd) including their different types with cardinalities.

Definition 3.5 Let ${\overset{˙}{G}}_{D}=(A,B)$ be an IFDG defined on an hidden digraph ${\overset{ˆ}{G}}_{D}$ = (V,E). Then

(*i*) $N_{E}(y)=\{z\in V:arc(y,z)\text{ is an effective arc}\}$ is an ENbhd of y∈V while $N_{E}[y]=N_{E}(y)\cup \{y\}$ is its CNbhd.

(ii)$N_{\lambda E}(y)$ = {z∈V: arc(y,z) is a semi *λ*- effective arc} is a semi *λ*- effective neighborhood of y∈V and $N_{\lambda E}[y]$ = $N_{\lambda E}(y)\cup \{y\}$ is the closed neighborhood of *y*.

(iii)$N_{\nu E}(y)$ = {z∈V:arc(y,z) is semi *ν*- effective arc} is a semi *ν*- effective neighborhood of y∈V and closed neighborhood of *y* is $N_{\nu E}[y]=N_{\nu E}(y)\cup \{y\}$.

(iv)$\delta_{E}({\overset{˙}{G}}_{D})$ = min$\left\{N_{E}(y):y\in V({\overset{˙}{G}}_{D})\right\}$ is the minimum cardinality of effective neighborhood.

(*v*) $\Delta_{E}({\overset{˙}{G}}_{D})$ = max $\left\{N_{E}(y):y\in V({\overset{˙}{G}}_{D})\right\}$ is the maximum cardinality of effective neighborhood.

Definition 3.6 Let y,z be any two vertices of an IFDG ${\overset{˙}{G}}_{D}$. If a directed edge (y,z) is an effective edge, then *y* dominates *z*.

Example 3.7 The directed edge (w,u) is an effective edge of an IFDG shown in Fig. 1 while the edges (u,v) and (v,w) are not the effective arcs. Thus the vertex *w* dominates *u* as there is an effective edge between these two vertices. However, a vertex *u* does not dominate *v*, and a vertex *v* does not dominate *w* as there are no effective edges between them.

Definition 3.8 Let ${\overset{˙}{G}}_{D}=(A,B)$ be an IFDG defined on an hidden digraph ${\overset{ˆ}{G}}_{D}$ = (V,E). A C⊂V is said to be a DS in an IFDG, if for each z∈V−C ∃ y∈C such that *y* dominates *z*.

Definition 3.9 If a dominating set *C* doesn't contain any other DS in an IFDG, then we call it a MDS. The minimum fuzzy cardinality among all DSs of an IFDG is said to be the effective arc domination number (DN), represented by $\omega_{E}({\overset{˙}{G}}_{D})$, and the corresponding DS is known as te minimum effective edge DS. The total members in the minimum effective edge DS is given by $n[\omega_{E}({\overset{˙}{G}}_{D})]$.

**Figure 2:**
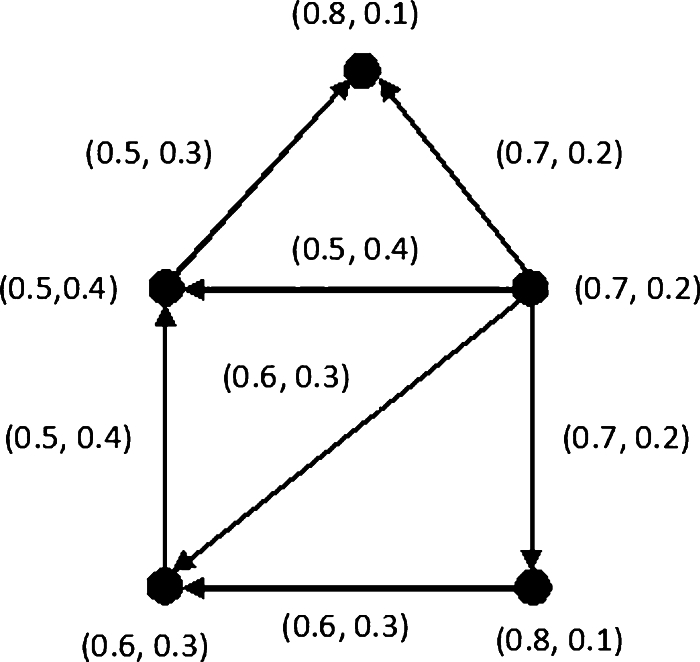
Minimal dominating sets.

Example 3.10 One can easily deduce that {(0.8,0.1),(0.7,0.2)}, {(0.5,0.4),(0.7,0.2)} and {(0.5,0.4),(0.8,0.1),(0.8,0.1)} are the MDSs of an IFDG ${\overset{˙}{G}}_{D}$ shown in Fig. 2.

Remark 3.11 Let ${\overset{˙}{G}}_{D}=(A,B)$ be an IFDG. If $\lambda_{B}(yz)<min(\lambda_{A}(y),\lambda_{A}(z))$, and $\nu_{B}(yz)>max(\nu_{A}(y)$,

$\nu_{A}(z))$, then $\left(\lambda_{A},\nu_{A}\right)$ is the only DS of ${\overset{˙}{G}}_{D}$.

**Figure 3:**
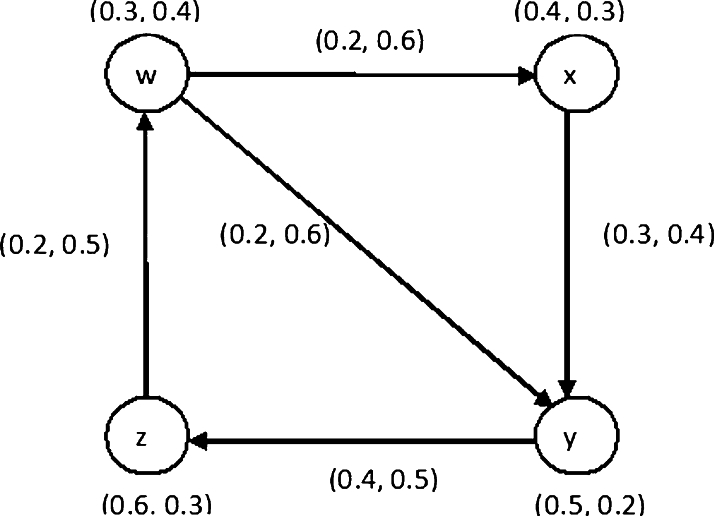
Dominating set in IFDG.

Example 3.12 By doing simple calculations, one can easily verify that {(0.3, 0.4), (0.4, 0.3), (0.5, 0.2), (0.6, 0.3)} is the only DS of an IFDG ${\overset{˙}{G}}_{D}$ shown in Fig. 3.

Definition 3.13 Let ${\overset{˙}{G}}_{D}$ be an IFDG and y,z be two vertices of ${\overset{˙}{G}}_{D}$. Then

(*i*) If the arc (y,z) is a semi *λ*- effective arc, then we say that *y* (semi *λ*- effective) dominates *z*.

(ii) If an arc (y,z) is a semi *ν*- effective arc, then *y* (semi *ν*- effective) dominates *z*.

Example 3.14 From Fig. 1, it can be easily seen that the arc (u,v) is semi-*ν* effective arc, hence *u* (semi *ν*- effective) dominate *v*. Similarly, the arc (v,w) is semi-*λ* effective arc, hence *v*(semi *λ*- effective) dominate *w*.

Remark 3.15 Let ${\overset{˙}{G}}_{D}$ be an IFDG and y,z be two vertices of ${\overset{˙}{G}}_{D}$. Then

(*i*) the total members in the minimum semi *λ*-effective arc ($\gamma_{\lambda E}(G)$) is the semi *λ*- effective arc DN, and is written as $n[\gamma_{\lambda E}(G)]$.

(ii) the total members in the minimum semi *ν*-effective arc ($\gamma_{\nu E}(G)$) is called the semi *ν*- effective arc DN, represented by $n[\gamma_{\nu E}(G)]$.

Theorem 3.16 *Let* ${\overset{˙}{G}}_{D}=(A,B)$ *be an IFDG of an hidden digraph* $G_{D}=(V,E)$ *. If* C⊂A *, then* $\sum_{x\in C}(\lambda_{A}(x),\nu_{A}(x))\leq |C|$ *, where* |C| *is the cardinality of C.*

Proof Since $0\leq (\lambda_{A}(x),\nu_{A}(x))\leq 1$, for all x∈C, then

$$\sum_{x\in C}(\lambda_{A}(x),\nu_{A}(x))\leq \sum_{x\in C}1=\sum_{m=1}^{\left|C\right|}1=1|C|=|C|.$$

 □

Theorem 3.17 *Let*${\overset{˙}{G}}_{D}=(A,B)$*be an IFDG and*$G_{D}=(V,E)$*be its hidden digraph with*S⊆V*. A DS*$\left(\lambda_{A}(S),\nu_{A}(S)\right)$*of*${\overset{˙}{G}}_{D}$*is minimal iff for each*$\left(\lambda_{A}(x),\nu_{A}(x))\in (\lambda_{A}(S),\nu_{A}(S)\right)$*, either*$\left(N_{{\overset{˙}{G}}_{D}})(\lambda_{A}(x),\nu_{A}(x))\cap (\lambda_{A}(S),\nu_{A}(S)\right)$*=* ∅ *or* $\left(N_{{\overset{˙}{G}}_{D}})(\lambda_{A}(y),\nu_{A}(y))\cap (\lambda_{A}(S),\nu_{A}(S))=\{\lambda_{A}(x),\nu_{A}(x)\right\}$*, for some* $\left(\lambda_{A}(y),\nu_{A}(y))\in (\lambda_{A},\nu_{A})\setminus (\lambda_{A}(S),\nu_{A}(S)\right)$*.*

Proof Let $\left(\lambda_{A}(x),\nu_{A}(x))\in (\lambda_{A}(S),\nu_{A}(S)\right)$ such that $\left(\lambda_{A}(S),\nu_{A}(S)\right)$ is a MDS of ${\overset{˙}{G}}_{D}$, then $\left(\lambda_{A}(S\right)$,

$\nu_{A}(S))\setminus (\lambda_{A}(x),\nu_{A}(x))$ is not a DS of ${\overset{˙}{G}}_{D}$. Hence $\left(\lambda_{A}(y),\nu_{A}(y))\notin (\lambda_{A}(S),\nu_{A}(S))\setminus (\lambda_{A}(x),\nu_{A}(x)\right)$ so that $\left(\lambda_{A}(y),\nu_{A}(y)\right)$ is not dominated by any of the member of $\left(\lambda_{A}(S),\nu_{A}(S))\setminus (\lambda_{A}(x),\nu_{A}(x)\right)$. Now we have the following cases.

Case 1. Let $\left(\lambda_{A}(y),\nu_{A}(y))=(\lambda_{A}(x),\nu_{A}(x)\right)$. Then $\left(\lambda_{A}(x),\nu_{A}(x)\right)$ is not dominated by any of the member of $\left(\lambda_{A}(S),\nu_{A}(S))\setminus (\lambda_{A}(x),\nu_{A}(x)\right)$ i.e.,$(N_{{\overset{˙}{G}}_{D}})((\lambda_{A}(x),\nu_{A}(x)))\cap (\lambda_{A}(S),\nu_{A}(S))=\emptyset$.

Case 2. Let $\left(\lambda_{A}(y),\nu_{A}(y))\neq (\lambda_{A}(x),\nu_{A}(x)\right)$. Then $\left(\lambda_{A}(y),\nu_{A}(y))\notin (\lambda_{A}(S),\nu_{A}(S)\right)$. Since $\left(\lambda_{A}(S\right)$,

$\nu_{A}(S))$ is a MDS of ${\overset{˙}{G}}_{D}$ implies $\left(\lambda_{A}(y),\nu_{A}(y)\right)$ is dominated by $\left(\lambda_{A}(x),\nu_{A}(x))\in (\lambda_{A}(S),\nu_{A}(S)\right)$. Thus $\left(N_{{\overset{˙}{G}}_{D}})(\lambda_{A}(y),\nu_{A}(y))\cap (\lambda_{A}(S),\nu_{A}(S))=\{\lambda_{A}(x),\nu_{A}(x)\right\}$, for some $\left(\lambda_{A}(y),\nu_{A}(y))\in (\lambda_{A},\nu_{A})\setminus (\lambda_{A}(S),\nu_{A}(S)\right)$.

Similarly, we can also proof its converse. □

Definition 3.18 An arrangement of effective arcs in which the starting node of subsequent edge is same as the ending node of previous edge is known as IF-directed path (IF-dipath) $P_{D}$.

Remark 3.19 Let $P_{D}=(A,B)$ be an IF-dipath of an hidden dipath $P_{n}=(V,C)$, where *n* represents an integer with m≥2. Then

1. $\left(\lambda_{A},\nu_{A}\right)$ = $\{(\lambda_{A}(x_{j}),\nu_{A}(x_{j})):x_{j}\in A$, for all j∈{1,2,.....,m}}.

2. $\left(\lambda_{B},\nu_{B}\right)$ =$\{(\lambda_{B}(x_{j},x_{j+1}),\nu_{B}(x_{j},x_{j+1})):(x_{j},x_{j+1})\in A$, for all j∈{1,2,...,(m−1)}}.

3. $\left(\lambda_{B}(x_{j},x_{j+1}),\nu_{B}(x_{j},x_{j+1})\right)$ = $\left(\lambda_{A}(x_{j}),\nu_{A}(x_{j})),(\lambda_{A}(x_{j+1}),\nu_{A}(x_{j+1})\right)$, for all j∈{1,2,...,(m−1)}.

4. In nontrivial IF-dipath, the first and last vertices are $\left(\lambda_{A}(x_{1}),\nu_{A}(x_{1})\right)$ and $\left(\lambda_{A}(x_{1}),\nu_{A}(x_{m})\right)$.

Theorem 3.20 *Let* $P_{D}$ *be an IF-dipath. Then one of the given conditions must be satisfied.*

1*.* $\gamma (P_{D})=\Sigma_{i=1}^{m/2}(\lambda_{A}(x_{2i-1}),\nu_{A}(x_{2i-1}))$

2*.* $\gamma (P_{D})=min$*X, where*

$$X=\{\sum_{i=1}^{(m+1)/2-i}(\lambda_{A}(x_{2i-1}),\nu_{A}(x_{2i-1}))+\sum_{i=(m+3)/2-j}^{(m+1)/2}(\lambda_{A}(x_{2i-2}),\nu_{A}(x_{2i-2})):j\in \{0,1,2,...,(m-1)/2\}\}.$$

Proof By Remark 3.19, $\left(\lambda_{A},\nu_{A})=\{(\lambda_{A}(x_{j}),\nu_{A}(x_{j})):x_{j}\in A,\forall j\in \{1,2,.....,m\}\right\}$ and

$$(\lambda_{B},\nu_{B})=\{(\lambda_{B}(x_{j},x_{j+1}),\nu_{B}(x_{j},x_{j+1})):(x_{j},x_{j+1})\in A,\forall j\in \{1,2,.....,(m-1)\}\}.$$

Because $\left(\lambda_{B}(x_{j},x_{j+1}),\nu_{B}(x_{j},x_{j+1})\right)$ is an effective edge for all j∈{1,2,...,(m−1)}, it implies $\left(\lambda_{A}(x_{j}),\nu_{A}(x_{j})\right)$ dominate $\left(\lambda_{A}(x_{j+1}),\nu_{A}(x_{j+1})\right)$, for all j∈{1,2,...,(m−1)}.

Case1:

If m=2i i.e., even, for positive integer *i*. Then

$$\left\{(\lambda_{A}(x_{1}),\nu_{A}(x_{1})),(\lambda_{A}(x_{3}),\nu_{A}(x_{3})),...,(\lambda_{A}(x_{m-1}),\nu_{A}(x_{m-1}))\right\}$$

is clearly the MDS of $P_{D}$. Note that

$$(\lambda_{A}(x_{1}),\nu_{A}(x_{1})),(\lambda_{A}(x_{3}),\nu_{A}(x_{3})),...,(\lambda_{A}(x_{m-1}),\nu_{A}(x_{m-1}))=\sum_{i=1}^{m/2}(\lambda_{A}(x_{2i-1}),\nu_{A}(x_{2i-1})).$$

Thus $\gamma (P_{D})=\Sigma_{i=1}^{m/2}(\lambda_{A}(x_{2i-1}),\nu_{A}(x_{2i-1}))$. This proves the statement (1).

Case2:

If m=2i−1 i.e., *m* is odd, for some integer *i*. Consequently,

$$\left\{(\lambda_{A}(x_{1}),\nu_{A}(x_{1})),(\lambda_{A}(x_{3}),\nu_{A}(x_{3})),...,(\lambda_{A}(x_{m}),\nu_{A}(x_{m}))\};\{(\lambda_{A}(x_{1}),\nu_{A}(x_{1})),(\lambda_{A}(x_{3}),\nu_{A}(x_{3})),...,(\lambda_{A}(x_{m-2}),\nu_{A}(x_{m-2})),(\lambda_{A}(x_{m-1}),\nu_{A}(x_{m-1}))\};\{(\lambda_{A}(x_{1}),\nu_{A}(x_{1})),(\lambda_{A}(x_{3}),\nu_{A}(x_{3})),...,(\lambda_{A}(x_{m-4}),\nu_{A}(x_{m-4})),(\lambda_{A}(x_{m-3}),\nu_{A}(x_{m-3})),(\lambda_{A}(x_{m-1}),\nu_{A}(x_{m-1}))\};.....\{(\lambda_{A}(x_{1}),\nu_{A}(x_{1})),(\lambda_{A}(x_{3}),\nu_{A}(x_{3})),...,(\lambda_{A}(x_{m-5}),\nu_{A}(x_{m-5})),(\lambda_{A}(x_{m-3}),\nu_{A}(x_{m-3})),(\lambda_{A}(x_{m-1}),\nu_{A}(x_{m-1}))\right\}$$

are MDSs of $P_{D}$.

Note that

$$(\lambda_{A}(x_{1}),\nu_{A}(x_{1})),(\lambda_{A}(x_{3}),\nu_{A}(x_{3})),...,(\lambda_{A}(x_{m-1}),\nu_{A}(x_{m-1}))=\sum_{i=1}^{(m+1)/2}(\lambda_{A}(x_{2i-1}),\nu_{A}(x_{2i-1})).$$

In general, we have

$$(\lambda_{A}(x_{1}),\nu_{A}(x_{1}))+(\lambda_{A}(x_{3}),\nu_{A}(x_{3}))+...+(\lambda_{A}(x_{k}),\nu_{A}(x_{k}))+(\lambda_{A}(x_{k+1}),\nu_{A}(x_{k+1}))+...+(\lambda_{A}(x_{m-3}),\nu_{A}(x_{m-3}))+(\lambda_{A}(x_{m-1}),\nu_{A}(x_{m-1}))=\sum_{i=1}^{(k+1)/2}(\lambda_{A}(x_{2i-1}),\nu_{A}(x_{2i-1}))+\sum_{i=(k+3)/2}^{(m+1)/2}(\lambda_{A}(x_{2i-2}),\nu_{A}(x_{2i-2})).$$

Let (j+1)/2+=(m+1)/2 for i∈{0,1,2,......(m−1)/2}. Then $\left(\lambda_{A}(x_{1}),\nu_{A}(x_{1}))+(\lambda_{A}(x_{3}),\nu_{A}(x_{3}))+...+(\lambda_{A}(x_{k}),\nu_{A}(x_{k}))+(\lambda_{A}(x_{k+1}),\nu_{A}(x_{k+1}))+...+(\lambda_{A}(x_{m-3}),\nu_{A}(x_{m-3}))+(\lambda_{A}(x_{m-1}\right)$,

$$\nu_{A}(x_{m-1}))=\sum_{i=1}^{(m+1)/2-j}(\lambda_{A}(x_{2i-1}),\nu_{A}(x_{2i-1}))+\sum_{i=(m+3)/2-j}^{(m+1)/2}(\lambda_{A}(x_{2i-2}),\nu_{A}(x_{2i-2})).$$

Thus the minimum of

$$X=\{\sum_{i=1}^{(m+1)/2-j}(\lambda_{A}(x_{2i-1}),\nu_{A}(x_{2i-1}))+\sum_{i=(m+3)/2-j}^{(m+1)/2}(\lambda_{A}(x_{2i-2}),\nu_{A}(x_{2i-2})):j\in \{0,1,2,.....,(m-1)/2\}\}$$

is the DN of a IF-dipath $P_{D}$. Therefore, $\gamma (P_{D})=minX$. □

**Figure 4:**
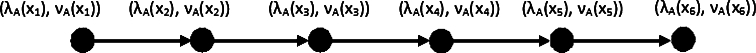
IF-directed path.

Example 3.21 Let $P_{D}=(A,B)$ be an IF-dipath of an hidden dipath $P_{6}=(V,C)$. Then from Fig. 4, the MDS of $P_{D}$ is $\left\{(\lambda_{A}(x_{1}),\nu_{A}(x_{1})),(\lambda_{A}(x_{3}),\nu_{A}(x_{3})),(\lambda_{A}(x_{5}),\nu_{A}(x_{5}))\right\}$ and the DN is $\gamma (P_{D})=\sum_{i=1}^{3}(\lambda_{A}(x_{2i-1}),\nu_{A}(x_{2i-1}))$.

**Figure 5:**
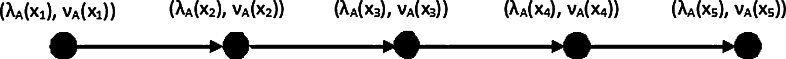
IF-directed path.

Example 3.22 From Fig. 5, let $P_{D}=(A,B)$ be a IF-dipath of an hidden dipath $P_{5}=(V,C)$. Let

$$P=\{(\lambda_{A}(x_{1}),\nu_{A}(x_{1})),(\lambda_{A}(x_{3}),\nu_{A}(x_{3})),(\lambda_{A}(x_{5}),\nu_{A}(x_{5}))\}Q=\{(\lambda_{A}(x_{1}),\nu_{A}(x_{1})),(\lambda_{A}(x_{3}),\nu_{A}(x_{3})),(\lambda_{A}(x_{5}),\nu_{A}(x_{5}))\}R=\{(\lambda_{A}(x_{1}),\nu_{A}(x_{1})),(\lambda_{A}(x_{3}),\nu_{A}(x_{3})),(\lambda_{A}(x_{5}),\nu_{A}(x_{5}))\}$$

Then MDS of $P_{D}$ is {P,Q,R} and the DN is

$$\gamma (P_{D})=min\{\sum_{(\lambda_{A}(x),\nu_{A}(x))\in P}(\lambda_{A}(x),\nu_{A}(x)),\sum_{(\lambda_{A}(y),\nu_{A}(y))\in Y}(\lambda_{A}(y),\nu_{A}(y)),\sum_{(\lambda_{A}(z),\nu_{A}(z)\in Z}(\lambda_{A}(z),\nu_{A}(z))\}.$$

Theorem 3.23 *Let* $P_{D}=(A,B)$ *be an IF-dipath of an hidden dipath* $P_{N}=(V,C)$ *. If* $\left(\lambda_{A}(x),\nu_{A}(x)\right)$

$=(\lambda_{A}(y),\nu_{A}(y))$*, then*$\frac{m}{2}(\lambda_{A}(x),\nu_{A}(x))$*,* ∀ x,y∈V*.*

Proof If *n* is even, then by Theorem 3.20, we have

$\gamma (P_{D})=\Sigma_{i=1}^{n/2}(\lambda_{A}(x_{2i-1}),\nu_{A}(x_{2i-1}))$. Since $\left(\lambda_{A}(x),\nu_{A}(x))=(\lambda_{A}(y),\nu_{A}(y)\right)$, ∀ x,y∈V, implies

$\left(\lambda_{A}(x_{1}),\nu_{A}(x_{1}))=(\lambda_{A}(x_{3}),\nu_{A}(x_{3}))=.....=(\lambda_{A}(x_{m-1}),\nu_{A}(x_{m-1}))=(\lambda_{A}(x),\nu_{A}(x)\right)$. Thus $\gamma (P_{D})=\sum_{k=1}^{m/2}(\lambda_{A}(x),\nu_{A}(x))=\frac{n}{2}(\lambda_{A}(x),\nu_{A}(x))$. Similarly, if *m* is odd then by Theorem 3.20, we have

$$\gamma (P_{D})=\sum_{i=1}^{(m+1)/2-j}(\lambda_{A}(x_{2i-1})),\nu_{A}(x_{2i-1}))+\sum_{i=(m+3)/2-j}^{(m+1)/2}(\lambda_{A}(x_{2i-2}),\nu_{A}(x_{2i-2})),\;\text{for all}\;j\in \{0,1,2,.....m\},(m-1)/2=\sum_{i=1}^{(m+1)/2-j}(\lambda_{A}(x),\nu_{A}(x))+\sum_{i=(m+3)/2-j}^{(m+1)/2}(\lambda_{A}(x),\nu_{A}(x))\;=((m+1)/2-j)(\lambda_{A}(x),\nu_{A}(x))+[(m+1)/2-((m+3)/2-j+1)](\lambda_{A}(x),\nu_{A}(x))\;=\frac{m+1}{2}(\lambda_{A}(x),\nu_{A}(x)).$$

Hence $\gamma (P_{D})$ is either $\frac{m}{2}(\lambda_{A}(x),\nu_{A}(x))$, if *m* is even or $\frac{m+1}{2}(\lambda_{A}(x),\nu_{A}(x))$, if *m* is odd. Consequently, $\gamma (P_{D})=\frac{m}{2}(\lambda_{A}(x),\nu_{A}(x))$. □

Definition 3.24 An IF-dicycle (directed cycle) $C_{D}$ is a dipath in which the starting and ending vertex is same.

Remark 3.25 Let $C_{D}=(A,B)$ be an IF-dicycle of an hidden dipath $P_{m}=(V,C)$, where *m* represents an integer with m≥3. Then

1. $\left(\lambda_{A},\nu_{A})=\{(\lambda_{A}(x_{j}),\nu_{A}(x_{j})):x_{j}\in A,\forall j\in \{1,2,.....,m\}\right\}$.

2. $\left(\lambda_{B},\nu_{B})=\{(\lambda_{B}(x_{j},x_{j+1}),\nu_{B}(x_{j},x_{j+1})),(\lambda_{B}(x_{m},x_{1}),\nu_{B}(x_{m},x_{1})):(x_{j},x_{j+1}),(x_{m},x_{1})\in A,\forall j\in \{1,2,.....,(m-1)\}\right\}$.

3. $\left(\lambda_{B}(x_{j},x_{j+1}),\nu_{B}(x_{j},x_{j+1}))=(\lambda_{A}(x_{j}),\nu_{A}(x_{j})),(\lambda_{A}(x_{j+1}),\nu_{A}(x_{j+1}))\forall j\in \{1,2,.....,(m-1)\right\}$.

Theorem 3.26 *Let* $C_{D}=(A,B)$ *be a IF-dicycle of an hidden dipath* $C_{m}=(V,C)$ *, where* m≥3 *. Then one of the given condition must be hold.*

1*.* $\gamma (C_{D})=min\{\Sigma_{i=1}^{m/2}(\lambda_{A}(x_{2i-1}),\nu_{A}(x_{2i-1})),\Sigma_{i=1}^{m/2}(\lambda_{A}(x_{2i}),\nu_{A}(x_{2i}))\}$*;*

2*.* $\gamma (C_{D})=min(X⋃Y)$*, where*

$$X=\{\sum_{i=1}^{(m+1)/2-j}(\lambda_{A}(x_{2i-1}),\nu_{A}(x_{2i-1}))+\sum_{i=(n+3)/2-j}^{(m+1)/2}(\lambda_{A}(x_{2i-2}),\nu_{A}(x_{2i-2})):j\in \{0,1,2,...,(m-1)/2\}\};$$

$$Y=\{\sum_{i=1}^{(m+1)/2-j}(\lambda_{A}(x_{2i}),\nu_{A}(x_{2i}))+\sum_{i=(m+3)/2-j}^{(m+1)/2}(\lambda_{A}(x_{2i-1}),\nu_{A}(x_{2i-1})):j\in \{0,1,2,...,(m-1)/2\}\}.$$

Proof Let I={1,2,...,m}. Because $C_{D}$ is a dicycle, the arcs $\left(\lambda_{B}(x_{j},x_{j+1}),\nu_{B}(x_{j},x_{j+1})\right)$, ∀ j∈I and $\left(\lambda_{B}(x_{m},x_{1}),\nu_{B}(x_{m},x_{1})\right)$ are effective. It implies that $\left(\lambda_{A}(x_{j}),\nu_{A}(x_{j})\right)$ dominate $\left(\lambda_{A}(x_{j+1}),\nu_{A}(x_{j+1})\right)$, for all j∈{1,2,...,m−1} and $\left(\lambda_{A}(x_{m}),\nu_{A}(x_{m})\right)$ dominate $\left(\lambda_{A}(x_{1}),\nu_{A}(x_{1})\right)$.

Case 1. If m=2i i.e., *m* is even, for any integer *i*. Then the given collection

$$\left\{(\lambda_{A}(x_{1}),\nu_{A}(x_{1})),(\lambda_{A}(x_{3}),\nu_{A}(x_{3})),...,(\lambda_{A}(x_{m-1}),\nu_{A}(x_{m-1}))\right\}$$

and

$$\left\{(\lambda_{A}(x_{2}),\nu_{A}(x_{2})),(\lambda_{A}(x_{4}),\nu_{A}(x_{4})),...,(\lambda_{A}(x_{m}),\nu_{A}(x_{m}))\right\}$$

are the DS as well as the MDSs of $C_{D}$. Note that

$$\left\{(\lambda_{A}(x_{1}),\nu_{A}(x_{1}))+(\lambda_{A}(x_{3}),\nu_{A}(x_{3}))+...+(\lambda_{A}(x_{m-1}),\nu_{A}(x_{m-1}))\}=\{\sum_{i=1}^{m/2}(\lambda_{A}(x_{2i-1}),\nu_{A}(x_{2i-1}))\right\}$$

and

$$\left\{(\lambda_{A}(x_{2}),\nu_{A}(x_{2}))+(\lambda_{A}(x_{4}),\nu_{A}(x_{4}))+...+(\lambda_{A}(x_{m}),\nu_{A}(x_{m}))\}=\{\sum_{i=1}^{m/2}(\lambda_{A}(x_{2i}),\nu_{A}(x_{2i}))\right\}$$

Thus $\gamma (C_{D})=min\{\Sigma_{i=1}^{m/2}(\lambda_{A}(x_{2i-1}),\nu_{A}(x_{2i-1})),\Sigma_{i=1}^{m/2}(\lambda_{A}(x_{2i}),\nu_{A}(x_{2i}))\}$. This proves the condition (1).

Case 2. If m=2i−1 i.e., *m* is odd, for any integer *i*. Then, we have the sets

$$\{(\lambda_{A}(x_{1}),\nu_{A}(x_{1})),(\lambda_{A}(x_{3}),\nu_{A}(x_{3})),...,(\lambda_{A}(x_{m}),\nu_{A}(x_{m}))\};$$

$$\{(\lambda_{A}(x_{1}),\nu_{A}(x_{1})),(\lambda_{A}(x_{3}),\nu_{A}(x_{3})),...,(\lambda_{A}(x_{m-2}),\nu_{A}(x_{m-2})),(\lambda_{A}(x_{m-1}),\nu_{A}(x_{m-1}))\};$$

$$\{(\lambda_{A}(x_{1}),\nu_{A}(x_{1})),(\lambda_{A}(x_{3}),\nu_{A}(x_{3})),...,(\lambda_{A}(x_{m-4}),\nu_{A}(x_{m-4})),(\lambda_{A}(x_{m-3}),\nu_{A}(x_{m-3})),$$

$$(\lambda_{A}(x_{m-1}),\nu_{A}(x_{m-1}))\};...\{(\lambda_{A}(x_{1}),\nu_{A}(x_{1})),(\lambda_{A}(x_{3}),\nu_{A}(x_{3})),...,(\lambda_{A}(x_{m-5}),\nu_{A}(x_{m-5})),$$

$$\left(\lambda_{A}(x_{m-3}),\nu_{A}(x_{m-3})),(\lambda_{A}(x_{m-1}),\nu_{A}(x_{m-1}))\right\}$$

are some of the DS as well as the MDSs of $C_{D}$.

Note that

$$(\lambda_{A}(x_{1}),\nu_{A}(x_{1}))+(\lambda_{A}(x_{3}),\nu_{A}(x_{3}))+...+(\lambda_{A}(x_{m}),\nu_{A}(x_{m}))=\sum_{i=1}^{(m+1)/2}(\lambda_{A}(x_{2i-1}),\nu_{A}(x_{2i-1})).$$

In general,

$$(\lambda_{A}(x_{1}),\nu_{A}(x_{1}))+(\lambda_{A}(x_{3}),\nu_{A}(x_{3}))+.....+(\lambda_{A}(x_{k}),\nu_{A}(x_{k}))+(\lambda_{A}(x_{k+1}),$$

$$\nu_{A}(x_{k+1}))+.......+(\lambda_{A}(x_{m-3}),$$

$$\nu_{A}(x_{m-3}))+(\lambda_{A}(x_{m-1}),\nu_{A}(x_{m-1}))$$

$$=\sum_{i=1}^{(k+1)/2}(\lambda_{A}(x_{2ki-1}),$$

$$\nu_{A}(x_{2i-1}))+\sum_{i=(k+3)/2}^{(m+1)/2}(\lambda_{A}(x_{2i-2}),\nu_{A}(x_{2i-2})).$$

Let (k+1)/2+j=(m+1)/2. Then

$$\sum_{i=1}^{(m+1)/2-j}(\lambda_{A}(x_{2i-1}),\nu_{A}(x_{2i-1}))+\sum_{i=(m+3)/2-j}^{(m+1)/2}(\lambda_{A}(x_{2i-2}),\nu_{A}(x_{2i-2})),\forall j\in \{0,1,2,.....,(m-1)/2\}.$$

Further to this, the sets

$$\{(\lambda_{A}(x_{2}),\nu_{A}(x_{2})),(\lambda_{A}(x_{4}),\nu_{A}(x_{4})),...,(\lambda_{A}(x_{m-1}),\nu_{A}(x_{m-1})),(\lambda_{A}(x_{m}),\nu_{A}(x_{m}))\};$$

$$\{(\lambda_{A}(x_{2}),\nu_{A}(x_{2})),(\lambda_{A}(x_{4}),\nu_{A}(x_{4})),...,(\lambda_{A}(x_{m-3}),\nu_{A}(x_{m-3})),(\lambda_{A}(x_{m-2}),\nu_{A}(x_{m-2})),$$

$$(\lambda_{A}(x_{m}),\nu_{A}(x_{m}))\};$$

$$\{(\lambda_{A}(x_{2}),\nu_{A}(x_{2})),(\lambda_{A}(x_{4}),\nu_{A}(x_{4})),...,(\lambda_{A}(x_{m-5}),\nu_{A}(x_{m-5})),$$

$$(\lambda_{A}(x_{m-4}),\nu_{A}(x_{m-4})),(\lambda_{A}(x_{m-2}),\nu_{A}(x_{m-2})),(\lambda_{A}(x_{m}),\nu_{A}(x_{m}))\};...,\{(\lambda_{A}(x_{2}),\nu_{A}(x_{2})),$$

$$\left(\lambda_{A}(x_{3}),\nu_{A}(x_{3})),...,(\lambda_{A}(x_{m-4}),\nu_{A}(x_{m-4})),(\lambda_{A}(x_{m-2}),\nu_{A}(x_{m-2})),(\lambda_{A}(x_{m}),\nu_{A}(x_{m})\right\}$$

are other DSs as well as the MDSs of $C_{D}$.

In general,

$$\{(\lambda_{A}(x_{2}),\nu_{A}(x_{2}))+(\lambda_{A}(x_{4}),\nu_{A}(x_{4}))+...+(\lambda_{A}(x_{k}),\nu_{A}(x_{k}))+(\lambda_{A}(x_{k+1}),\nu_{A}(x_{k+1}))+.......+(\lambda_{A}(x_{m-2}),$$

$$\nu_{A}(x_{m-2}))+(\lambda_{A}(x_{m}),\nu_{A}(x_{m}))\}=\sum_{i=1}^{(k)/2}(\lambda_{A}(x_{2i}),\nu_{A}(x_{2i}))+\sum_{i=(k+2)/2}^{(m+1)/2}(\lambda_{A}(x_{2i-1}),\nu_{A}(x_{2i-1})).$$

Let k/2+j=(m+1)/2. Then

$$\{(\lambda_{A}(x_{2}),\nu_{A}(x_{2}))+(\lambda_{A}(x_{4}),\nu_{A}(x_{4}))+...+(\lambda_{A}(x_{k}),\nu_{A}(x_{k}))+(\lambda_{A}(x_{k+1}),\nu_{A}(x_{k+1}))+.......+(\lambda_{A}(x_{m-2}),$$

$$\nu_{A}(x_{m-2}))+(\lambda_{A}(x_{m}),\nu_{A}(x_{m}))\}=\sum_{i=1}^{(m+1)/2-j}(\lambda_{A}(x_{2i}),\nu_{A}(x_{2i}))+\sum_{i=(m+3)/2-j}^{(m+1)/2}(\lambda_{A}(x_{2i-1}),\nu_{A}(x_{2i-1})),\;\forall j\in \{0,1,2,.....,(m-1)/2\}.$$

Let $J^{\prime }=\{0,1,2,...,(m-1)/2\}$ with

$$X=\{\sum_{i=1}^{(m+1)/2-j}(\lambda_{A}(x_{2i-1}),\nu_{A}(x_{2i-1}))+\sum_{i=(m+3)/2-j}^{(m+1)/2}(\lambda_{A}(x_{2i-2}),\nu_{A}(x_{2i-2})):j\in J^{\prime }\};$$

$$Y=\{\sum_{i=1}^{(m+1)/2-j}(\lambda_{A}(x_{2i}),\nu_{A}(x_{2i}))+\sum_{i=(m+3)/2-j}^{(m+1)/2}(\lambda_{A}(x_{2i-1}),\nu_{A}(x_{2i-1})):j\in J^{\prime }\}.$$

Hence, the DN of $C_{D}$ is $\gamma (C_{D})=min(X⋃Y)$. Hence condition (2) is satisfied. □

**Figure 6:**
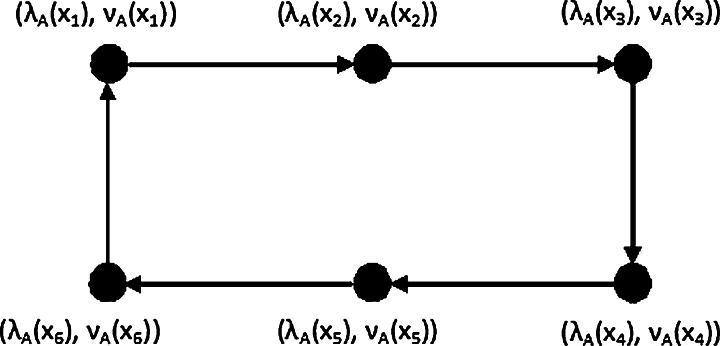
IF-directed cycle.

Example 3.27 Let $C_{D}=(A,B)$ be a IF-dicycle of an hidden dicycle $C_{6}=(V,C)$. Let $X=\{(\lambda_{A}(x_{1}),\nu_{A}(x_{1})),(\lambda_{A}(x_{3}),\nu_{A}(x_{3})),(\lambda_{A}(x_{5}),\nu_{A}(x_{5}))\}$,

and $Y=\{(\lambda_{A}(x_{2}),\nu_{A}(x_{2})),(\lambda_{A}(x_{4}),\nu_{A}(x_{4})),(\lambda_{A}(x_{6}),\nu_{A}(x_{6}))\}$, (see Fig. 6) The MDS of $C_{D}$ is {X,Y} and the DN is

$\gamma (C_{D})=min\{\sum_{i=1}^{3}(\lambda_{A}(x_{2i-1}),\nu_{A}(x_{2i-1}),\sum_{i=1}^{3}(\lambda_{A}(x_{2i}),\nu_{A}(x_{2i})))\}$.

Example 3.28 Let $C_{D}=(A,B)$ be a IF dicycle of an hidden dicycle $C_{5}=(V,C)$. Let

$$X_{1}=\{(\lambda_{A}(x_{1}),\nu_{A}(x_{1})),(\lambda_{A}(x_{3}),\nu_{A}(x_{3})),(\lambda_{A}(x_{5}),\nu_{A}(x_{5}))\},$$

$$X_{2}=\{(\lambda_{A}(x_{1}),\nu_{A}(x_{1})),(\lambda_{A}(x_{2}),\nu_{A}(x_{2})),(\lambda_{A}(x_{4}),\nu_{A}(x_{4}))\},$$

$$X_{3}=\{(\lambda_{A}(x_{2}),\nu_{A}(x_{2})),(\lambda_{A}(x_{3}),\nu_{A}(x_{3})),(\lambda_{A}(x_{5}),\nu_{A}(x_{5}))\},$$

$$X_{4}=\{(\lambda_{A}(x_{1}),\nu_{A}(x_{1})),(\lambda_{A}(x_{3}),\nu_{A}(x_{3})),(\lambda_{A}(x_{4}),\nu_{A}(x_{4}))\},$$

$$X_{5}=\{(\lambda_{A}(x_{2}),\nu_{A}(x_{2})),(\lambda_{A}(x_{4}),\nu_{A}(x_{4})),(\lambda_{A}(x_{5}),\nu_{A}(x_{5}))\}.$$

**Figure 7:**
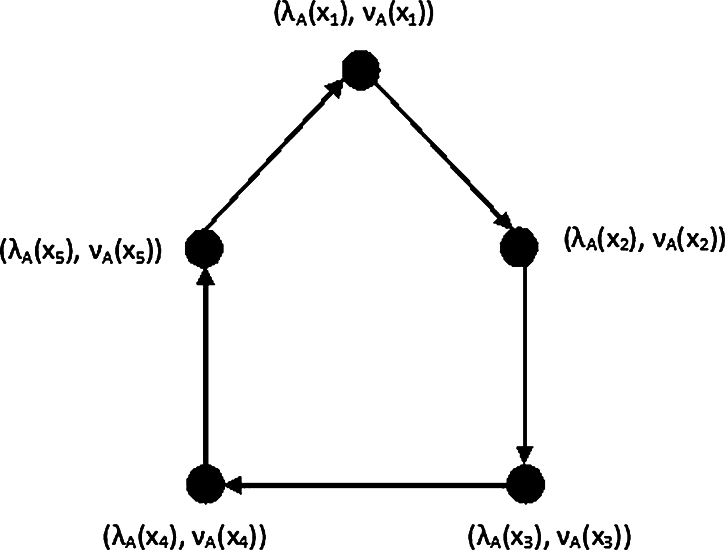
IF-directed cycle.

Then from Fig. 7, the MDS of $C_{D}$ is $\left\{X_{i}:1,2,.....,5\right\}$ and the DN is $\gamma (C_{D})=min\{\sum_{\{}(\lambda_{A}(x),\nu_{A}(x))\in X_{i}(\lambda_{A}(x),\nu_{A}(x):j=1,2,.....5)\}$.

Theorem 3.29 *Let* $C_{D}=(A,B)$ *be a IF dicycle of an hidden dicycle* $C_{n}=(V,C)$ *, where*

n≥3 *. If* $\left(\lambda_{A}(x),\nu_{A}(x))=(\lambda_{A}(y),\nu_{A}(y)\right)$ *, for all* x,y∈V *, then* $\left[\frac{m}{2}](\lambda_{A}(x),\nu_{A}(x)\right)$ *.*

Proof If *n* is even, by Theorem 3.26

$$\gamma (C_{D})=min\{\sum_{i=1}^{m/2}(\lambda_{A}(x_{2i-1}),\nu_{A}(x_{2i-1})),\sum_{i=1}^{m/2}(\lambda_{A}(x_{2i}),\nu_{A}(x_{2i}))\}.$$

Because $\left(\lambda_{A}(x),\nu_{A}(x))=(\lambda_{A}(y),\nu_{A}(y)\right)$, for all x,y∈V, it implies

$$\gamma (C_{D})=\sum_{i=1}^{m/2}(\lambda_{A}(x),\nu_{A}(x))=(\frac{m}{2})(\lambda_{A}(x),\nu_{A}(x))$$

using the same reasons as in Theorem 3.26.

Similarly, if *m* is odd, by Theorem 3.26

$$\sum_{i=1}^{\frac{m+1}{2}-j}(\lambda_{A}(x_{2i-1}),\nu_{A}(x_{2i-1}))+\sum_{i=(m+3)/2-j}^{(m+1)/2}(\lambda_{A}(x_{2i-2}),\nu_{A}(x_{2i-2})),\forall j\in \{0,1,2,.....,(m-1)/2\}=\sum_{i=1}^{\frac{m+1}{2}-j}(\lambda_{A}(x),\nu_{A}(x))+\sum_{i=(m+3)/2-j}^{(m+1)/2}(\lambda_{A}(x),\nu_{A}(x))$$

Because

$$(\lambda_{A}(x),\nu_{A}(x))=(\lambda_{A}(y),\nu_{A}(y)),\forall x,y\in V,\;=((m+1)/2-j)(\lambda_{A}(x),\nu_{A}(x))+[(m+1)/2-((m+3)/2-j+1)](\lambda_{A}(x),\nu_{A}(x))\;=(\frac{m+1}{2})(\lambda_{A}(x),\nu_{A}(x)).$$

and

$$\sum_{i=1}^{(m+1)/2-j}(\lambda_{A}(x_{2i}),\nu_{A}(x_{2i}))+\sum_{i=(m+3)/2-j}^{(m+1)/2}(\lambda_{A}(x_{2i-1}),\nu_{A}(x_{2i-1})),\forall j\in \{0,1,2,.....,(m-1)/2\}\;=\sum_{i=1}^{(m+1)/2-j}(\lambda_{A}(x),\nu_{A}(x))+\sum_{i=(m+3)/2-j}^{(m+1)/2}(\lambda_{A}(x),\nu_{A}(x)),$$

since

$$(\lambda_{A}(x),\nu_{A}(x))=(\lambda_{A}(y),\nu_{A}(y)),\forall x,y\in V,=(\frac{m+1}{2})(\lambda_{A}(x),\nu_{A}(x)).$$

Hence, $\gamma (C_{D})$ is either $\left(\frac{m}{2})(\lambda_{A}(x),\nu_{A}(x)\right)$ if *m* is even, or $\left(\frac{m+1}{2})(\lambda_{A}(x),\nu_{A}(x)\right)$ if *m* is odd.

Thus $\gamma (C_{D})=[\frac{m}{2}](\lambda_{A}(x),\nu_{A}(x))$. □

## 4 Application of domination in IFDGs through effective arcs

Numerous FGs have been used to model the daily life issues related to communication networks, selection criterions etc. Here, we present the model based on dominations in IFDGs to resolve the issue related to the selection of suitable places for opening new restaurants. For the sake of comparative study, first we model the scenario by using the concepts of domination in FDGs and then we investigate it by the concepts of dominations in IFDGs. We notice that dealing with such problem by using our presented model is more consistent and relatable in comparison with the former one.

### 4.1 Model through FDG

**Figure 8:**
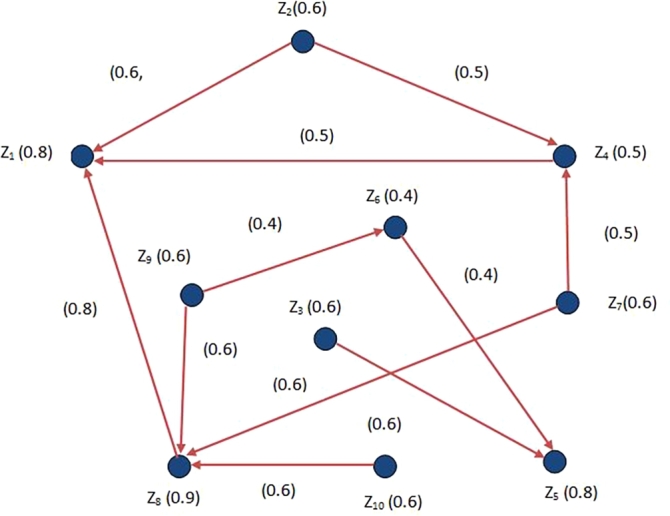
Model through FDG.

Suppose an owner of restaurant likes to open different restaurants in distinct locations of the cities. He cannot open restaurants in all locations because of his budget. In different locations, he wants to open minimum number of restaurants with maximum profit so that maximum number of people in the city can explore the restaurants. He gets the maximum profit, if he opens the restaurants in the hustling areas of cities where most of the peoples want to explore the restaurants. In FDG which is shown in Fig. 8, the sights are considered as nodes and their MD indicate the percentages of peoples who want to visit the restaurant. Directed edges represent the directed (one way) roads between the cities, and their MD denote the percentages of peoples who want to eat the restaurant's food. One can easily check that in Fig. 8, the arcs $\left(Z_{2},Z_{1}),(Z_{2},Z_{4}),(Z_{4},Z_{1}),(Z_{8},Z_{1}),(Z_{9},Z_{8}),(Z_{9},X_{6}),(Z_{6},Z_{5}),(Z_{3},Z_{5}),(Z_{10},Z_{8}),(Z_{7},Z_{8}\right)$ and $\left(Z_{7},Z_{4}\right)$ are effective arcs and the set $\left\{Z_{1},Z_{5},Z_{8}\right\}$ is a MDS. Following Table 2, these areas have MD, so 80% people of city $Z_{1}$, 80% people of city $Z_{5}$ and 90% people of city $Z_{8}$ wants to visit the restaurant and number of roads connecting (directly linked) to these areas in comparison to the other areas are larger in number. So, $Z_{1}$, $Z_{5}$ and $Z_{8}$ are optimal locations to open the restaurants.

**Table 2:** Data set.

*Vertices*	*percentage of peoples visited the restaurants*
*Z*_1_	0.8
*Z*_2_	0.6
*Z*_3_	0.6
*Z*_4_	0.5
*Z*_5_	0.8
*Z*_6_	0.4
*Z*_7_	0.6
*Z*_8_	0.9
*Z*_9_	0.6
*Z*_10_	0.6

In the above model, the surveyor didn't count the likings and disliking of the peoples. Consequently, the outcome obtained from this model will not be more useful.

### 4.2 Model through IFDG

**Figure 9:**
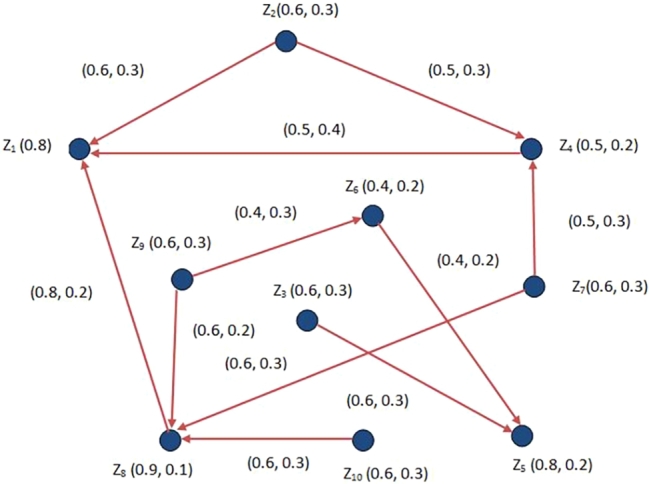
Model through IFDG.

As in the model based on FDG, the liking and disliking of the community cannot be properly described, so we use the concept of IFDG to obtain the appropriate outcomes as compared to that of the model of FDG. In Fig. 9 IFDG is shown, where the MD of the nodes represent the peoples who wants to visit the restaurant food and the NMD represent the percentage of people who do not wants to visit the restaurant. In IFDG, the directed arcs (direct routes) represent the percentages of peoples who like to eat the restaurant's food. These values have been obtained by conducting a survey of the residents living in surrounding areas of the proposed location. To deal with this problem, we utilize the idea of domination in IFDGs based on effective arcs. The arcs $\left(Z_{2},Z_{1}),(Z_{2},Z_{4}),(Z_{4},Z_{1}),(Z_{8},Z_{1}),(Z_{9},Z_{8}),(Z_{9},Z_{6}),(Z_{6},Z_{5}),(Z_{3},Z_{5}),(Z_{10},Z_{8}),(Z_{7},Z_{8}\right)$ and $\left(Z_{7},Z_{4}\right)$ are the effective arcs. We find the DS of IFDG given in Fig. 9. In this way, it would help us to open the minimum number of restaurants and hence to provide food at all locations nearby city. By a simple calculation, we can observe that the set $\left\{Z_{1},Z_{5},Z_{8}\right\}$ is the MDS. From Table 3, these locations almost have the greater MD 80% people of city $Z_{1}$, 80% people of city $Z_{5}$ and 90% people of city $Z_{8}$ wants to visit the restaurant and 20% people of city $Z_{1}$, 20% people of city $Z_{5}$ and 10% people of city $Z_{8}$ will not visit the restaurant and also the roads which connect these areas in comparison with the other sights are larger in numbers. Hence the best places to open the restaurants are $Z_{1}$, $Z_{5}$ and $Z_{8}$. Since the MD of $Z_{2}$ and $Z_{4}$ is 60% and 50%, then 60% and 50% residents of these locations could visit the restaurant at $Z_{1}$ and degrees of NMD of $Z_{2}$ and $Z_{4}$ are 30% and 20%, then 30% and 20% residents of such areas will not be able to visit the restaurant at $Z_{1}$. Since MD of $Z_{3}$ and $Z_{6}$ is 60% and 40%, then 60% and 40% residents of such points can visit the restaurant at $Z_{5}$, the NMD of $Z_{3}$ and $Z_{6}$ are 30% and 20%, then 30% and 20% resident belonging to these points will not visit the restaurant at $Z_{5}$. Since the degree of MD of all cities at $Z_{7}$, $Z_{9}$ and $Z_{10}$ is 60%, 60% residents of such points can come to restaurant at $Z_{8}$ and the NMD value of all cities at $Z_{7}$, $Z_{9}$ and $Z_{10}$ is 30%, then 30% residents living in these places will not visit the restaurant at $Z_{8}$ as shown in the Fig. 9. If the restaurant are opened in these areas, then the owner will achieve a maximum profit and consequently he will earn more. Moreover, we also provide a pseudo-code for proposed algorithm based on domination in IFDGs through which we can easily obtain the solution of our targeted problem.

**Table 3:** Data set.

*Vertices*	*percentage of peoples visited*	*percentage of peoples not visited*
	*restaurants*	*restaurants*
*Z*_1_	0.8	0.2
*Z*_2_	0.6	0.3
*Z*_3_	0.6	0.3
*Z*_4_	0.5	0.2
*Z*_5_	0.8	0.2
*Z*_6_	0.4	0.2
*Z*_7_	0.6	0.3
*Z*_8_	0.9	0.1
*Z*_9_	0.6	0.3
*Z*_10_	0.6	0.3

**Pseudo-code:**

Input: IFS of restaurants A

Output: MDS

void FMDS()

IFS A = getIFSOfRestaurants();

int no of Restaurants = count(A);

IFS B;

Set DS;

for (int x = 0; x <no of Restaurants; x++)

for (int y = 0; y <no of Restaurants; y++)

if (A (x,y))

$\lambda_{B}(x,y)$ = min$\left(\lambda_{A}(x),\lambda_{A}\right)$;

$\nu_{B}(x,y)$ =max$\left(\nu_{A}(x),\nu_{A}(y))\right)$;

if (arc is effective(x, y, B))

DS.add(x);

Set MDS = find MDS(DS);

boolarc is effective(int x, int y, IFS B)

return $(\lambda_{B}(x,y)>\lambda$ threshold) ∧ $(\nu_{B}(xy)<\nu$ threshold);

struct IFS

map < pair < int, int >, double>*λ*;

map< pair < int, int >, double >*ν*;

boolisAdjacent(int x, int y);;

IFS getIFSOfRestaurants()

IFS A;

return A;

int count(IFS A)

return $\lambda_{A}$.size();

## 5 Comparative analysis

The notion of domination in fuzzy FGs hss its own importance, many problems in real-world containing uncertainties have been dealt through the concept of domination in FGs. The concept of FGs was introduced by Rosenfeld and has been further generalized in many ways. FGs uses membership degree for vertices and edges under specific condition. Many problems have been solved using fuzzy graphs and effective results were obtained. FGs like FSs were generalized in different ways to model complex problem containing uncertainties. FDGs was the generalization of FGs, it uses FSs to express values for vertices and edges. Both FGs and FDGs express data using membership value and receive much attention of the researchers. However, with each day going on uncertainties in problem also getting complex. Hence there is a need of new flexible structure to achieve accurate outcomes. Consequently, the idea of IFGs was introduced by using IFSs i.e., MD and NMD were used for vertices and edges. The notion of domination was also initiated for IFGs. To deal with uncertain problems with more accuracy, the idea of IFDGs was also presented. Moreover, our presented notion of domination in IFDGs by utilizing effective arcs is comparatively more useful than the other generalization of FGs exiting in literature such as FDGs, IFGs etc. As an evidence, if we deal the situation given in the above application through FDGs, then $X_{4}$ is 90% effective in opening the restaurants. Consequently, it has only MD which provides a limited representation of best place to open the restaurants. Thus, it becomes one sided as ineffectiveness is disregarded. As a result, the overall effectiveness of best place to open the restaurants may be inaccurately assessed. To overcome this situation one can study such problems through IFDGs which utilizes MD and NMD values to determine the MDSs. In this way, one can express the percentage of the effectiveness as well as the ineffectiveness in order to opening the restaurants. As we have demonstrated in the above application that MDSs are helpful in selecting the best place for opening the restaurants. Hence our model approach is more generalized and flexible, providing a vast space for modeling and analyzing uncertain information across different domains. Moreover, we also provide a characteristic comparison of our proposed model with FDGs and IFDGs in Table 4.

**Table 4:** The characteristic comparison of IFDGs with FDGs.

Characteristics	Fuzzy directed graphs	Intuitionistion Fuzzy directed graphs
Representation	Membership values for the	Membership value for the
	effectiveness for the	effectiveness and the
	best place to open	non-membership values for
	the restaurants	ineffectiveness

Effectiveness for the	Limited view,	Balanced view,
best place to open	focuses only on	considers both
the restaurants	effectiveness	effectiveness
		and ineffectiveness

Proficiency	Limited proficiency	Better proficiency
	as a result of	as a result of
	focusing only on	focusing on effectiveness
	effectiveness	and ineffectiveness

Flexibility in	Limited flexibility	More flexible
representation		
of MDSs.		

## 6 Conclusion and future perspectives

In this article, we have introduced the notion of domination in IFDG, by utilizing effective arcs we have provided numerous important results and its application. Domination in FDG based on strong arcs was already explored but the concepts of domination in IFDG with effective arcs was not discussed in literature. Therefore, we have addressed the gap existing in literature regarding domination. Initially, we initiated many typed of effective arcs in IFDG including semi-*λ* effective arcs, semi-*ν* effective arcs. Subsequently, we introduced the notions of domination related to IFDG by utilizing on effective arcs and discussed its several significant characteristics. Along with some fascinating results, we have described DS and MDS. We have also introduced the concept of IF-dipath and IF-dicycle in IFDGs and investigated their domination numbers. Additionally, we have provided their MDSs and DNs along with various fascinating results. Through application, we have investigated that the domination in IFDG produces efficient results compared to FDGs. In this regard, we have investigated the problem of opening restaurants in several areas of city based on both the dominations in FDGs and IFDGs. For future studies, one could extend the concepts presented in this study to bipolar fuzzy directed graphs, complex fuzzy directed graphs, Pythagorean fuzzy graphs etc.

## CRediT authorship contribution statement

**Waheed Ahmad Khan:** Writing – review & editing, Writing – original draft, Validation, Methodology, Investigation, Formal analysis, Conceptualization. **Khadija Ali:** Writing – original draft, Methodology, Investigation, Formal analysis, Conceptualization. **Amna Fida:** Writing – review & editing, Writing – original draft, Validation, Methodology, Investigation, Formal analysis, Conceptualization. **Muhammad Asif:** Writing – review & editing, Writing – original draft, Methodology, Investigation, Conceptualization. **Hai Van Pham:** Writing – review & editing, Validation, Methodology, Investigation, Conceptualization. **Quoc Hung Nguyen:** Writing – review & editing, Writing – original draft, Investigation, Formal analysis, Conceptualization. **Thanh Trung Le:** Writing – review & editing, Investigation, Formal analysis, Conceptualization. **Le Phuc Thinh Tran:** Writing – review & editing, Writing – original draft, Methodology, Investigation, Formal analysis, Conceptualization.

## Declaration of Competing Interest

The authors declare that they have no known competing financial interests or personal relationships that could have appeared to influence the work reported in this paper.

## Data Availability

No any data set is used in this research.
