# Corrigendum to “Some novel concepts of intuitionistic fuzzy directed graphs with application in selecting a suitable place for opening restaurant” [Heliyon Volume 10, Issue 14, July 2024, Article e33950]

**DOI:** 10.1016/j.heliyon.2025.e43228

**Published:** 2025-03-20

**Authors:** Waheed Ahmad Khan, Khadija Ali, Amna Fida, Muhammad Asif, Hai Van Pham, Quoc Hung Nguyen, Thanh Trung Le, Le Phuc Thinh Tran

**Affiliations:** a Division of Science and Technology, Department of Mathematics, University of Education Lahore, Attock Campus, Attock, 43600, Pakistan; b Division of Science and Technology, Department of Computer Science, University of Education Lahore, Attock Campus, Attock, 43600, Pakistan; c School of Information and Communication Technology, Hanoi University of Science and Technology, Hanoi, Viet Nam; d School of Business Information Technology, University of Economics, Ho Chi Minh City, Viet Nam

In the original published version of this article, reference [10] was added in error:

***[10] C. Jana, H. Garg, M. Pal, Multi-attribute decision making for power dombi operators under pythagorean fuzzy information with mabac method, J. Ambient Intell. Humaniz. Comput. 14 (8) (2023) 10761–10778.***

The intext citation for this reference appears in the Introduction:

*Ubaid ur Rehman [9] introduced some aggregation operators under complex fuzzy environment and applied towards multi-attribute decision-making.****Jana et al. [10] initiated the notion of dombi operators under pythagorean fuzzy information and provided applications in multi-attribute decision making****. Mahapatra et al. [11] initiated the applications of edge coloring of FGs*.

Reference [10] and the line featuring the citation have now been removed:

*Ubaid ur Rehman [9] introduced some aggregation operators under complex fuzzy environment and applied towards multi-attribute decision-making. Mahapatra et al. [11] initiated the applications of edge coloring of FGs*.

The authors apologize for the errors.

## Declaration of competing interest

The authors declare that they have no known competing financial interests or personal relationships that could have appeared to influence the work reported in this paper.

